# Quantitative and qualitative assessment of airborne microorganisms during gross anatomical class and the bacterial and fungal load on formalin-embalmed corpses

**DOI:** 10.1038/s41598-024-69659-y

**Published:** 2024-08-17

**Authors:** Jonas Keiler, Antje Bast, Jessy Reimer, Markus Kipp, Philipp Warnke

**Affiliations:** 1 Institute of Anatomy, Rostock University Medical Center, Gertrudenstrasse 9, 18057 Rostock, Germany; 2 Institute for Medical Microbiology, Virology and Hygiene, Rostock University Medical Center, Schillingallee 35, 18057 Rostock, Germany

**Keywords:** Mold growth, Medical education, Human gross anatomy, Formaldehyde, Microbiology, Embalming, Anatomy, Air microbiology, Antifungal agents

## Abstract

Mold growth on body donations remains an underreported yet serious issue in anatomical teaching. Bacterial and fungal growth pose health risks to lecturers and students, alongside with ethical and aesthetic concerns. However, limited information exists on the presence of bacteria and fungi on body donations and their underlying causes. To investigate the potential impact of airborne germs on body donation contamination, we conducted indoor air measurements before, during, and after our anatomical dissection course, with outdoor measurements serving as a control. Tissue samples from the dissected body donations were collected to assess the germ load, with qualitative and quantitative microbiological analyses. Air samples from the dissection hall contained no fungi, but various fungal species were identified in the adjacent stairways and outdoors which implies that fungal occurrence in the dissection hall air was independent of lecturers’ and students’ presence. Moreover, our results indicate that adequate ventilation filters can effectively reduce indoor fungal germs during courses, while the bacterial load in room air appears to increase, likely due to the presence of lecturers and students. Additionally, the tissue samples revealed no bacterial or fungal germs which implies that our ethanol-formalin-based embalming solution demonstrates an effective long-term antimicrobial preservation of corpses.

## Introduction

Embalmed body donations play a crucial role in medical education, serving as a fundamental element in anatomical dissection courses^1–3^. These courses typically utilize bodies donated by recently deceased individuals, embalmed within 48 h post-mortem, primarily using a formaldehyde-based fixation solution^4,5^. The use of formaldehyde not only preserves tissue structures at both microscopic and macroscopic levels^6^, but also provides effective long-term anti-microbial preservation^7^ by eliminating the body’s endogenous microbiome and protecting against exogenous microbial contamination, ideally maintaining the body’s integrity until cremation.

During anatomical dissection courses, medical and paramedical students dissect human corpses to study the morphology, texture and topography of the human body. Typically, such a course lasts for several weeks, and several students work on the same cadaver to deepen their understanding of the human anatomy. Thus, a well effective body conservation is necessary to allow prolonged dissections. Mold growth on embalmed body donations presents a significant yet often overlooked challenge in anatomical education, as documented by various studies^4,8–11^. Fundamentally, it is challenging to entirely prevent mold formation on cadavers in the dissection room. Despite bodies being pre-treated with a fixative solution that reduces the microbial loads, students and lecturers invariably introduce pathogens, which consecutively might proliferate in or on the surface of the corpses. The growth of bacteria and fungi on donated bodies might pose health risks to instructors and students involved in dissections^12,13^ thus raising ethical concerns while decomposition and mold might cover anatomically relevant surface structures such as nerves or epifascial veins. Despite the significance of the issue, there is a notable lack of comprehensive data regarding the prevalence and causes of microbial growth on body donations and prevention possibilities.

The human microbiome is a dynamic, complex system with a unique composition, abundance, and distribution of microbial species throughout an individual’s lifetime^14^. Premortal pathologies can significantly modify certain components of this system^15^ whereas the postmortem thanatomicrobiome is shaped by a variety of endogenous and exogenous factors that play a role in human decomposition^16^.

Numerous studies have examined airborne germs across diverse settings, including office buildings and hospitals^17–21^. However, to the best of our knowledge, detailed insights into the air quality during anatomical dissection courses, especially in moderate climates, and comprehensive analyses on mold growth on body donations are lacking.

To investigate the potential influence of airborne germs on the contamination of embalmed body donations, impaction-based air sampling was performed, and indoor and outdoor germ concentrations were assessed before, during, and after an anatomical dissection course. Additionally, to evaluate the bacterial and fungal burden in the embalmed body donations, tissue samples were analyzed throughout the course.

## Results

### Temperature and humidity

**Figure 1:**
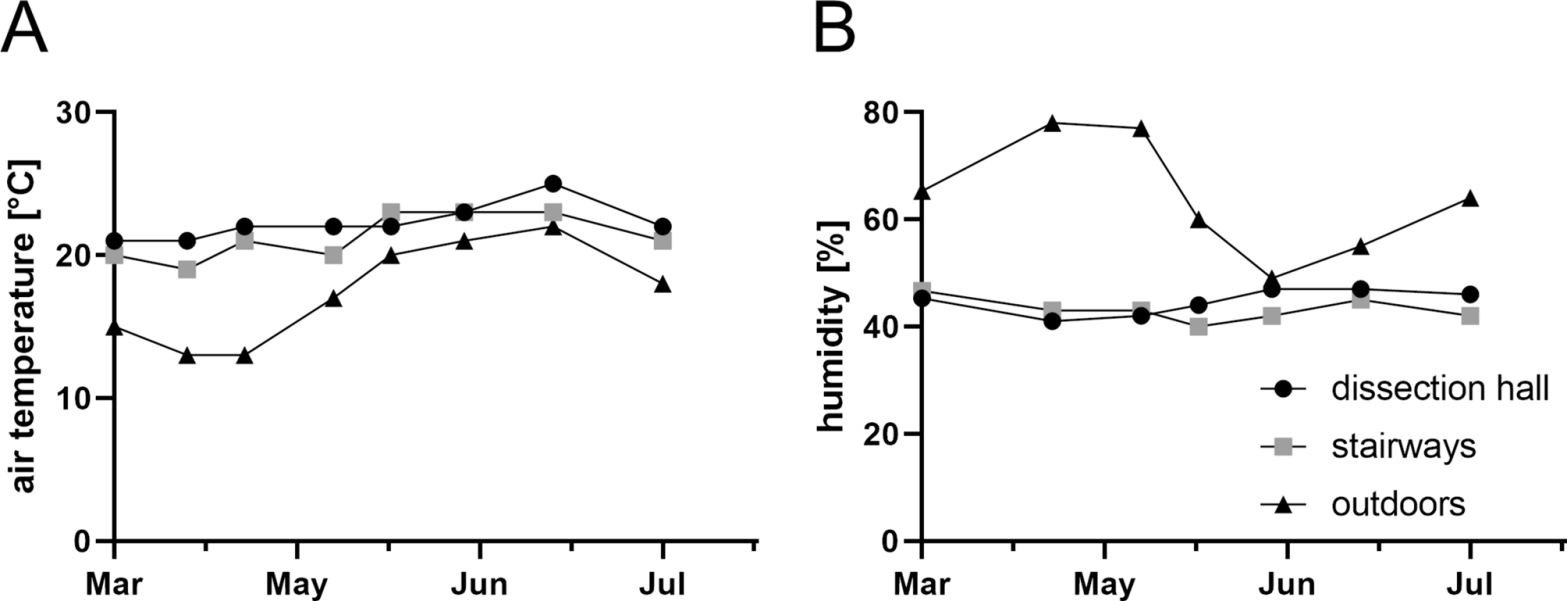
Temporal course of air temperature (**A**) and humidity (**B**) in dissection hall (circles), stairways (rectangles) and outdoors (triangles) during microbiological room air measurements*.* First and last measurements (in March and July, respectively) were done in the semester break before and after the course (without students being present in dissection hall and stairways).

Temperature and humidity at the indoor locations (dissection hall and stairways) were relatively stable in the study period between March and July. Mean indoor temperature (± standard deviation) was 22.3 ± 1.3 °C for the dissection hall and 21.3 ± 1.6 °C for the stairways. Mean indoor humidity (± standard deviation) was 44.6 ± 2.4% for the dissection hall and 43.1 ± 2.2% for the stairways. Temperature and humidity at the outdoor location altered over time, with a mean temperature of 17.4 ± 3.5 °C and 64.0 ± 10.7% humidity on average (Fig. 1).

### Microbiological air measurements

**Figure 2:**
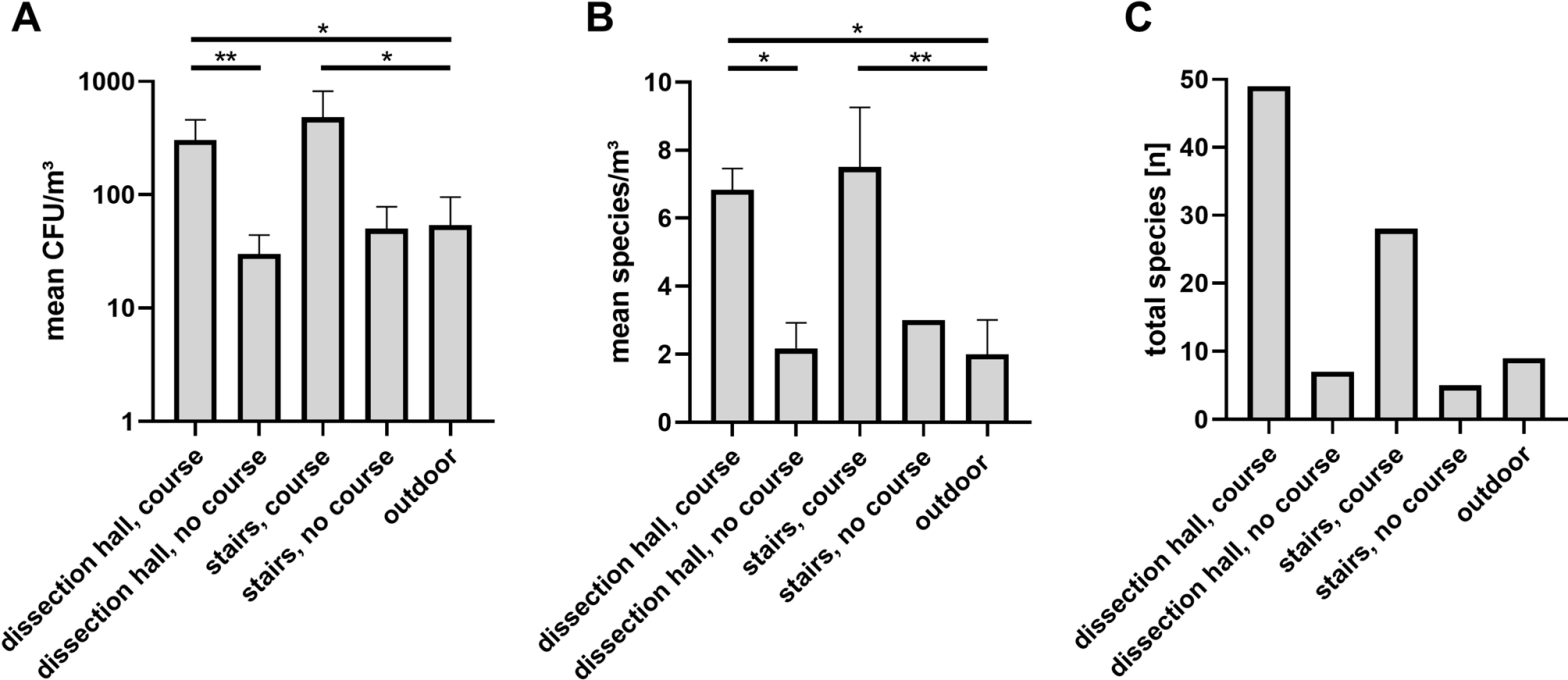
Bacterial load of the measured air in the different locations and at different time points: during course (with students) vs. no course (semester break, no students). Results are presented either as mean ± standard deviation (A,B) or as total numbers (C). For the dissection hall, all 3 individual measurement values are presented in A and B. Statistical significant differences (see also Table 2) are indicated for A and B. (**A**) Bacterial colony forming units (CFU) per m^3^. (**B**) Mean species per m^3^ room air**. **(**C**) Total species at the different locations and at different time points. *p ≤ 0.05, **p ≤ 0.005.

The highest mean concentrations of bacteria in the room air were found during the anatomical dissection course for the stairways adjacent to the dissection hall (481.7 ± 340.2 CFU/m^3^) and inside the dissection hall (305.6 ± 138.7 CFU/m^3^), while concentrations were significantly lower without the presence of students one week before and one week after the anatomical dissection course (50.0 ± 28.3 CFU/m^3^ in the stairways and 30.0 ± 14.1 CFU/m^3^ in the dissection hall). Bacterial germ concentrations during outdoor measurements were 54.0 ± 41.0 CFU/m^3^ on average and were significantly lower than in the measurements in dissection hall and stairways during the course (Tables 1–4; Fig. 2).

**Table 1:** Summary of mean bacterial colony forming units per m^3^ (CFU/m^3^) and mean fungal species numbers in the measured locations: the air outside the anatomy building (”outdoor”), inside the dissection hall during the course (i.e. with students; “course”), before and after the course semester (i.e. without students; ”semester break”), and inside the stairways during the course (i.e. with occasional students; ”course”), as well as before and after the course semester (i.e. without students; ”semester break”).

	Outdoor	Dissection hall, course^1^	Dissection hall, semester break (no students)^1^	Stairways, course	Stairways, semester break (no students)
mean CFU/m^3^ (bacteria)	54.0 ± 41.0	305.6 ± 138.7	30.0 ± 14.1	481.7 ± 340.2	50.0 ± 28.3
bacterial genera	7	23	5	15	3
mean bacterial genera	2.0 ± 1.0	8.7 ± 2.7	3.5 ± 0.7	5.2 ± 1.8	2.0 ± 1.4
bacterial species	8	47	7	28	5
mean bacterial species	2.0 ± 1.0	14.2 ± 2.5	2.2 ± 0.8	7.5 ± 1.8	3.0 ± 0.0
fungal genera	7	0	1	8	3
mean fungal genera	3.6 ± 1.5	0.0 ± 0.0	0.2 ± 0.4	2.2 ± 1.0	2.5 ± 2.1
fungal species	15	0	1	11	5
mean fungal species	3.6 ± 1.5	0.0 ± 0.0	0.2 ± 0.4	2.2 ± 1.0	2.5 ± 2.1

^1^includes the averaged value from all 3 measured locations in the dissection hall.

Means are given ± standard deviation.

**Table 2:** Comparison of the different locations and time points (during course vs. no course) concerning the mean bacterial colony forming units (CFU) and the mean bacterial species number per m^3^.

	Mean CFU/m^3^	Mean bacterial species/m^3^
	adjusted p value	adjusted p value
dissection hall, course vs. no course	0.0013	0.0413
dissection hall, course vs. outdoor	0.0249	0.0440
dissection hall, no course vs. outdoor	> 0.9999	> 0.9999
stairs, course vs. outdoor	0.0253	> 0.9999
stairs, no course vs. outdoor	> 0.9999	> 0.9999
stairs, course vs. no course	0.2286	0.6966
dissection hall, course vs. stairs, course	> 0.9999	0.0076
dissection hall, no course vs. stairs, no course	> 0.9999	> 0.9999

The Kruskal–Wallis test was performed for the analysis of variance (ANOVA). For selected multiple comparisons between location the Dunn’s post-hoc test was used.

Differences were considered significant at p ≤ 0.05.

**Table 3:** Colony forming units per m^3^ (CFU/m^3^) for the 10 most abundant bacterial genera in the air at all examined locations (mean ± standard deviation).

	Mean CFU/m^3^				
Bacterial genera	Outdoor	Dissection hall, course^1^	Dissection hall, semester break (no students)^1^	Stairways, course	Stairways, semester break (no students)
*Micrococcus*	2.0 ± 4.5	124.4 ± 55.4	6.7 ± 4.7	188.3 ± 105.7	0.0 ± 0.0
*Staphylococcus*	2.0 ± 4.5	122.2 ± 88.7	11.7 ± 7.1	180.0 ± 179.7	0.0 ± 0.0
*Bacillus*	2.0 ± 4.5	8.9 ± 18.6	0.0 ± 0.0	15.0 ± 36.7	0.0 ± 0.0
*Kocuria*	0.0 ± 0.0	5.6 ± 4.6	1.7 ± 2.4	8.3 ± 16.0	0.0 ± 0.0
*Pantoea*	0.0 ± 0.0	3.3 ± 5.6	0.0 ± 0.0	11.7 ± 28.6	15.0 ± 21.2
*Corynebacterium*	0.0 ± 0.0	2.8 ± 3.9	8.3 ± 11.8	5.0 ± 8.4	25.0 ± 7.1
*Neobacillus*	0.0 ± 0.0	1.1 ± 2.7	0.0 ± 0.0	23.3 ± 57.2	0.0 ± 0.0
*Dermacoccus*	0.0 ± 0.0	0.6 ± 1.4	0.0 ± 0.0	21.7 ± 53.1	0.0 ± 0.0
*Pseudarthrobacter*	22.0 ± 31.9	0.0 ± 0.0	0.0 ± 0.0	10.0 ± 16.7	0.0 ± 0.0
*Arthrobacter*	20.0 ± 23.5	0.0 ± 0.0	0.0 ± 0.0	0.0 ± 0.0	0.0 ± 0.0

Taxa are sorted by mean concentration in the room air of the dissection hall during the anatomical dissection course.

^1^includes the averaged values from all 3 measured locations in the dissection hall.

**Table 4:** Colony forming units per m^3^ (CFU/m^3^) for the 15 most abundant bacterial species in the air at all examined locations (mean ± standard deviation).

			Mean CFU/m^3^		
Bacterial species	Outdoor	Dissection hall, course^1^	Dissection hall, semester break (no students)^1^	Stairways, course	Stairways, semester break (no students)
*Micrococcus luteus*	0.0 ± 0.0	121.7 ± 54.1	6.7 ± 4.7	176.7 ± 113.1	0.0 ± 0.0
*Staphylococcus hominis*	0.0 ± 0.0	38.3 ± 43.3	6.7 ± 4.7	50.0 ± 81.5	0.0 ± 0.0
*Staphylococcus capitis*	0.0 ± 0.0	29.4 ± 23.8	1.7 ± 2.4	46.7 ± 60.9	0.0 ± 0.0
*Staphylococcus epidermidis*	0.0 ± 0.0	24.4 ± 29.8	0.0 ± 0.0	40.0 ± 59.0	0.0 ± 0.0
*Rothia mucosa*	0.0 ± 0.0	8.9 ± 14.4	0.0 ± 0.0	0.0 ± 0.0	0.0 ± 0.0
*Staphylococcus haemolyticus*	2.0 ± 4.5	8.9 ± 8.3	0.0 ± 0.0	13.3 ± 17.5	0.0 ± 0.0
*Staphylococcus succinus*	0.0 ± 0.0	6.1 ± 15.0	0.0 ± 0.0	13.3 ± 32.7	0.0 ± 0.0
*Moraxella osloensis*	0.0 ± 0.0	6.1 ± 7.7	1.7 ± 2.4	8.3 ± 9.8	0.0 ± 0.0
*Bacillus subtilis*	0.0 ± 0.0	5.6 ± 13.6	0.0 ± 0.0	13.3 ± 32.7	0.0 ± 0.0
*Micrococcus terreus*	0.0 ± 0.0	2.2 ± 5.4	0.0 ± 0.0	11.7 ± 28.6	0.0 ± 0.0
*Staphylococcus saprophyticus*	0.0 ± 0.0	2.2 ± 5.4	0.0 ± 0.0	8.3 ± 20.4	0.0 ± 0.0
*Neobacillus drentensis*	0.0 ± 0.0	1.1 ± 2.7	0.0 ± 0.0	23.3 ± 57.2	0.0 ± 0.0
*Dermacoccus nishinomiyaensis*	0.0 ± 0.0	0.6 ± 1.4	0.0 ± 0.0	21.7 ± 53.1	0.0 ± 0.0
*Pseudarthrobacter scleromae*	17.5 ± 35.0	0.0 ± 0.0	0.0 ± 0.0	6.7 ± 10.3	0.0 ± 0.0
*Arthrobacter koreensis*	18.0 ± 24.9	0.0 ± 0.0	0.0 ± 0.0	0.0 ± 0.0	0.0 ± 0.0

Species are sorted by mean concentration in the room air of the dissection hall during the anatomical dissection course.

^1^includes the averaged values from all 3 measured locations in the dissection hall.

A total of 60 bacterial species belonging to 31 genera were detected during the air measurements. The number of detected *taxa* was highest in the dissection hall when occupied with students during the anatomical course (a total of 23 genera and 47 species) while it was lower without students one week before and one week after the anatomical course (a total of 5 genera and 7 species). The outdoor measurements yielded a similarly low number of taxa (a total of 7 genera and 8 species).

Most bacterial species were assigned to *Staphylococcus* (n = 14), followed by *Corynebacterium* (n = 5) and *Bacillus* (n = 5). Three species each were assigned to *Kocuria* and *Pantoea,* whereas two species each were assigned to *Streptococcus*, *Pseudarthrobacter* and *Arthrobacter* (Supplementary Table S1).

In the outdoor measurements (n = 5), *Pseudarthrobacter* (22.0 ± 31.9 CFU/m^3^) and *Arthrobacter* (20.0 ± 23.5 CFU/m^3^) exhibited the highest average concentration, followed by *Staphylococcus*, *Micrococcus* and *Bacillus* (2.0 ± 4.5 CFU/m^3^ each), while other bacterial germs were not detected during our outdoor measurements.

One week before and one week after the anatomical course, when students were absent, bacterial germ concentrations were low in the dissection hall und the adjacent stairways. The highest bacterial germ concentrations during these measurements were observed for *Staphylococcus* (11.7 ± 7.1 CFU/m^3^), *Corynebacterium* (8.3 ± 11.8 CFU/m^3^) and *Micrococcus* (6.7 ± 4.7 CFU/m^3^) in the hall and *Corynebacterium* (25.0 ± 7.1 CFU/m^3^) and *Pantoea* (15.0 ± 21.2 CFU/m^3^) in the stairways.

During the anatomical course (with students and lecturers), germ concentrations in the dissection hall and the adjacent stairways were at their peak. The highest values were measured for *Micrococcus* (124.4 ± 55.4 CFU/m^3^ in the dissection hall, 188.3 ± 105.7 CFU/m^3^ in the stairways) and *Staphylococcus* (122.2 ± 88.7 CFU/m^3^ in the dissection hall, 180.0 ± 179.7 CFU/m^3^ in the stairways) and, with *Micrococcus luteus* showing the highest concentration (121.7 ± 54.1 CFU/m^3^ in the dissection hall, 176.7 ± 113.1 CFU/m^3^ in the stairways), followed by *Staphylococcus hominis* (38.3 ± 43.3 CFU/m^3^ in the dissection hall, 50.0 ± 81.5 CFU/m^3^ in the stairways), *Staphylococcus capitis* (29.4 ± 23.8 CFU/m^3^ in the dissection hall, 46.7 ± 60.9 CFU/m^3^ in the stairways) and *Staphylococcus epidermidis* (24.4 ± 29.8 CFU/m^3^ in the dissection hall, 40.0 ± 59.0 CFU/m^3^ in the stairways).

Twenty-three fungal species belonging to 11 genera (Table 5; Supplementary-Table S2) were identified. Most fungal species were detected outdoors and in the stairways with *Aspergillus niger* and *Aspergillus fumigatus* being predominantly present in both locations, followed by *Penicillium chrysogenum* and *Aspergillus puulaausensis* in the stairways. Throughout all 6 time points during the anatomical dissection course (with students and lecturers), no fungal species were detected at any of the 3 locations in the dissection hall. A similar scenario occurred during both time points when students were absent from the dissection hall (one week before and one week after the course), except for the detection of *Aspergillus niger* in one measurement at one of the 3 locations (referred to as “near blackboard”) within the hall. Of note, yeasts were not detected at all.

**Table 5:** Detected fungal genera in the different locations: the air outside the anatomy building (first column), inside the dissection hall during the course (i.e. with students; second column), before and after the course semester (i.e. without students; third column), and inside the stairways during the course (i.e. with occasional students; fourth column), as well as before and after the course semester (i.e. without students; fifth column).

	Detected fungal germs				
Genera	Outdoor [n = 5]	Dissection hall, course [n = 6]^1^	Dissection hall, semester break (no students) [n = 2]^1^	Stairways, course [n = 6]	Stairways, semester break (no students) [n = 2]
*Aspergillus*	+	−	+	+	+
*Penicillium*	+	−	−	+	+
*Alternaria*	+	−	−	+	−
*Talaromyces*	+	−	−	−	+
*Mollisia*	+	−	−	+	−
*Agrocybe*	−	−	−	+	−
*Purpureocillium*	−	−	−	+	−
*Ustilago*	+	−	−	−	−
*Arthrinium*	−	−	−	+	−
*Neurospora*	+	−	−	−	−
*Fomitopsis*	−	−	−	+	−

^1^presence for all 3 measured locations in the dissection hall was pooled.

### Microbial loads in tissues from embalmed body donations

No bacterial or fungal germs were detected after incubation of the tissue samples taken during the anatomical course from the human corpses.

## Discussion

Despite its allergenic and carcinogenic properties, formaldehyde remains a common agent for permanently erasing the thanatomicrobiom in embalming. However, its usage is often restricted by occupational safety regulations^22^.

Our embalmment solution contains a relatively low concentration of formaldehyde (2%), in line with recommendations from the German-Austrian-Swiss Anatomical Society^22^. This cautious approach is attributed to occupational safety measures and strict European Union regulations governing formaldehyde’s use as a biocide for preserving biological tissues and specimens. Our exemplary tissue samples showed no evidence of bacterial or fungal contamination, suggesting a largely sterile condition and effective long-term antimicrobial properties of our embalming solution, consistent with previous studies on similarly formulated solutions^23^. Possibly, the months-long immersion post-fixation in aqueous phenol might have an additional, antimicrobial effect. These findings differ from other studies which reported a microbial presence in formalin-embalmed corpses^24^, although other anatomical regions than in our study, such as, e.g., the axilla, oronasal region and perineum, were sampled. The detected species in these studies were assigned to, e.g., *Corynebacterium*, *Staphylococcus epidermidis* and *Staphylococcus hominis*.

Estimating the overall extent of mold growth on formaldehyde-embalmed cadavers used in anatomical education is challenging due to scarce data, which are predominantly found in publications with ambiguous scientific standards^8–11^. The presence of fungal germs in embalmed corpses may often go unnoticed due to a temporary lack of superficial conidial growth, despite the presence of deep mycelium. Similarly, the presence of bacterial germs is often not readily apparent and may therefore be underestimated^13,24^.

While the skeletal musculature in vivo is generally sterile under physiological conditions, the large intestine harbors the highest microbial diversity in the human body. The pre-mortem intestinal microbiome in healthy individuals consists mostly of fungi such as *Candida*, *Saccharomyces*, *Penicillium*, *Aspergillus*, *Cryptococcus*, *Malassezia*, *Cladosporium*, *Galactomyces*, *Debaryomyces* and *Trichosporon*. Some of these fungi can grow and colonize the gut, while others are acquired from diet or environment, such as *Penicillium* and *Aspergillus*^25,26^. Fungal abundance and diversity on the skin are reported to be low^27^, with *Malassezia* species being the most prevalent and *Candida* species being opportunistic fungal pathogens commonly found on the human cutis^28^.

Our findings suggest that our ethanol-formaldehyde-based embalming process not only eliminates presumably all endogenous intestinal germs but also prevents re-colonization and contamination by exogenous germs. This is particularly evident for airborne bacterial germs, given the high bacterial load during the dissection course. However, potential re-colonization by airborne fungal germs cannot be ruled out due to their absence in the dissection hall. In rare cases during past courses, we observed mold growth with *Aspergillus* or *Penicillium* species, but formalin-embalmed corpses remained unaffected. Nevertheless, we have occasionally observed unspecified mold growth on formalin-embalmed donation corpses in the past decade.

Possible sources of tissue contamination in body donations may include endogenous germs, contact^13^, or airborne transmission. The wearing of lab coats and gloves, as well as medical face masks or surgical caps and ventilating of filtered air might circumvent the latter factors.

In the ventilation system of our dissection hall, a filter of class ISO ePM1 ≥ 80% according to DIN EN ISO 16890–1 is used as the second filter stage immediately at the outlet of the ventilation unit. This filter class has a particle separation efficiency of at least 80% for particles ≤ 1 µm. Consequently, besides fungal germs, particles such as viruses, bacteria, nanoparticles, soot (from fossil fuels), sea salt, and oil mist are separated to at least 80%.

Based on our findings exhaled air does not appear to be a source of airborne fungal germs. However, these findings are illustrative due to the absence of non-embalmed tissue as a control, which presents challenges and ethical concerns when simultaneously applying fresh human tissue. Previous studies have shown that the mycobiome in air and dust is dominated by *Penicillium*, *Cladosporium*, *Aspergillus*, *Alternaria* and yeasts^29^, consistent with our findings from the analyzed outdoor air. *Aspergillus fumigatus*, which is potentially pathogenic for humans^30^ has been detected both outdoors and in the stairways during the course but not in the dissection hall, neither before, during or after the course. In our experience, students tend to leave the dissection hall to do pauses more often toward the end of the course, mostly due to advanced progress concerning the given dissection goals. This correlates with increased airborne germs in the stairways, which does not have a ventilation filter system, toward the end of the course semester (not shown).

Bacteria belonging to the microbiome of the oral cavity and the respiratory tract, or the skin were most abundant in our measurements, probably resulting from the individuals present during class. The measured mean indoor concentrations of bacterial germs in our study are similar to those reported in other studies^19^. Generally, mean indoor concentration of bacterial and fungal germs differ between summer and winter, with higher concentrations observed during summer attributed to favorable environmental factors for microbial growth and reproduction.

In conclusion, possible sources of tissue contaminations in body donations include endogenous germs that may have persisted despite body embalmment, contact or airborne transmission introduced through ventilated room air or direct contact with lecturers, tutors or students. Our findings indicate that the presence of fungal germs in the room air of our dissection hall is not influenced by the presence of lecturers, tutors and students rather than by ambient air entry. Therefore, in case of problems with mold grow on cadavers during courses, prevention measures should, in the first place, focus on air filtering systems. On the other hand, bacterial load in the room air appears to increase, likely due to the presence of lecturers, tutors and students. This might be of relevance since current embalmment research focusses on formalin-free fixative solutions due to the cancerogenic potential of formalin—with potentially less antibiotic effects as compared to conventional embalmment. So far, ethanol-based formalin embalming solution demonstrates effective long-term antimicrobial preservation of dissected corpses.

It is important to acknowledge limitations in our study. Since our tissue sampling for assessing microbial load was only exemplary and not exhaustive, certain germs may have gone undetected despite their presence in specific anatomical regions of the corpse. Furthermore, we did not evaluate the potential germ load in the ethanolic solution used to moisten the enveloping sheets around the body donations. Further extensive studies will be necessary to confirm our findings and their relationship with the potential germ load in the treatments of body donations.

## Methods

All methods were carried out in accordance with guidelines of the German Science Foundation (“Guidelines for Safeguarding Good Research Practice”).

### Anatomical dissection course

The study was conducted in the rooms of the Institute of Anatomy (Rostock University Medical Center; Germany) during the anatomical dissection course, held from April to June 2023. This study was approved by the Rostock University Medical Center ethics committee (approval ID: A 2016–0083) and conducted in accordance with the Declaration of Helsinki on the ethical principles for medical research involving human subjects from October 2013.

### Embalmment

Body donors gave their informed consent during lifetime to use their remains for research studies. Body donations were embalmed approximately 5–7 month prior to the anatomical dissection course. The embalming solution of approximately 6–7 L was administered between 1 and 4 days after death with an automatic pump via the femoral artery of the left thigh and comprised a mixture of 2% formaldehyde (Roth, Karlsruhe, Germany article# 7398.5) in 58.6% butanone-denatured ethanol (Walter CMP, Kiel, Germany, article# 6616025), 8.5% glycerol (Roth, article# 7533.4), 0.4% saturated thymol (Caelo, Hilden, Germany, article# 2692) and 0.3% saturated salicylic acid (Caelo, article# 2036). After transfemoral perfusion, the body donations were immersed for 4–6 month in 0.5% aqueous phenol for post-fixation.

After post-fixation, the body donations were placed on stainless dissection tables and enveloped in cotton sheets which were moistened with alcoholic glycerol (52% EtOH, 8% glycerol). The enveloped corpses were then ensheathed with a polyethylene foil to avoid desiccation of the body donation.

### Organization and infrastructural aspects of the anatomical dissection course

Two successive groups with approximately 110 individuals each, including lecturers, tutors, and students, each equipped with lab coat and nitrile gloves, occupied the dissection hall which had a room air volume of about 1000 m^3^. The hall was equipped with 16 dissection tables, each featuring a formalin-fixed body donation.

During the anatomical dissection course, the body donations were unveiled regularly 4–5 times per week and exposed to room air in supine or prone position during dissection for approximately 180 min each time. Before re-enveloping and depending on the degree of present moistness, cotton sheets were moistened again with alcoholic glycerol.

Students occasionally used the stairways adjacent to the hall, and the door between the stairways and the dissection hall was sporadically opened as students entered or exited. Another door, leading to an adjoining preparation room, was infrequently opened, primarily when lecturers needed access to or from the hall.

Inlet air was provided via air supply hoods which are suspended above each dissection table. Waste air was sucked off via near-ground ventilation trunks along the walls. The ventilation system in the dissection hall had a filter of class ISO ePM1 ≥ 80% according to DIN EN ISO 16890–1 which is used as the second filter stage immediately at the outlet of the ventilation unit. This filter class had a declared particle separation efficiency of at least 80% for particles ≤ 1 µm.

### Microbiology

Our microbiological laboratory is accredited by the Germany national accreditation authority (DAkkS) and regularly monitored by internal and external quality controls. The samples were handled according to the protocols based on DIN EN ISO 15189.

### Room air measurements

Room air measurements were conducted in 2023 at the Institute of Anatomy of the Rostock University Medical Center, Germany, at 6 time points during the anatomical dissection course (April 13th, April 24th, May 11th, May 22nd, June 5th, June 22nd) as well as one week before (March 20th) and one week after (July 13th) the anatomical dissection course. Investigators wore surgical masks and disinfected gloves during sampling.

During the study period, basic environmental parameters (temperature, relative humidity, air pressure) were measured repeatedly by a mobile station (Greininger electronic GmbH, GFTB 100, Regenstauf, Germany).

**Figure 3:**
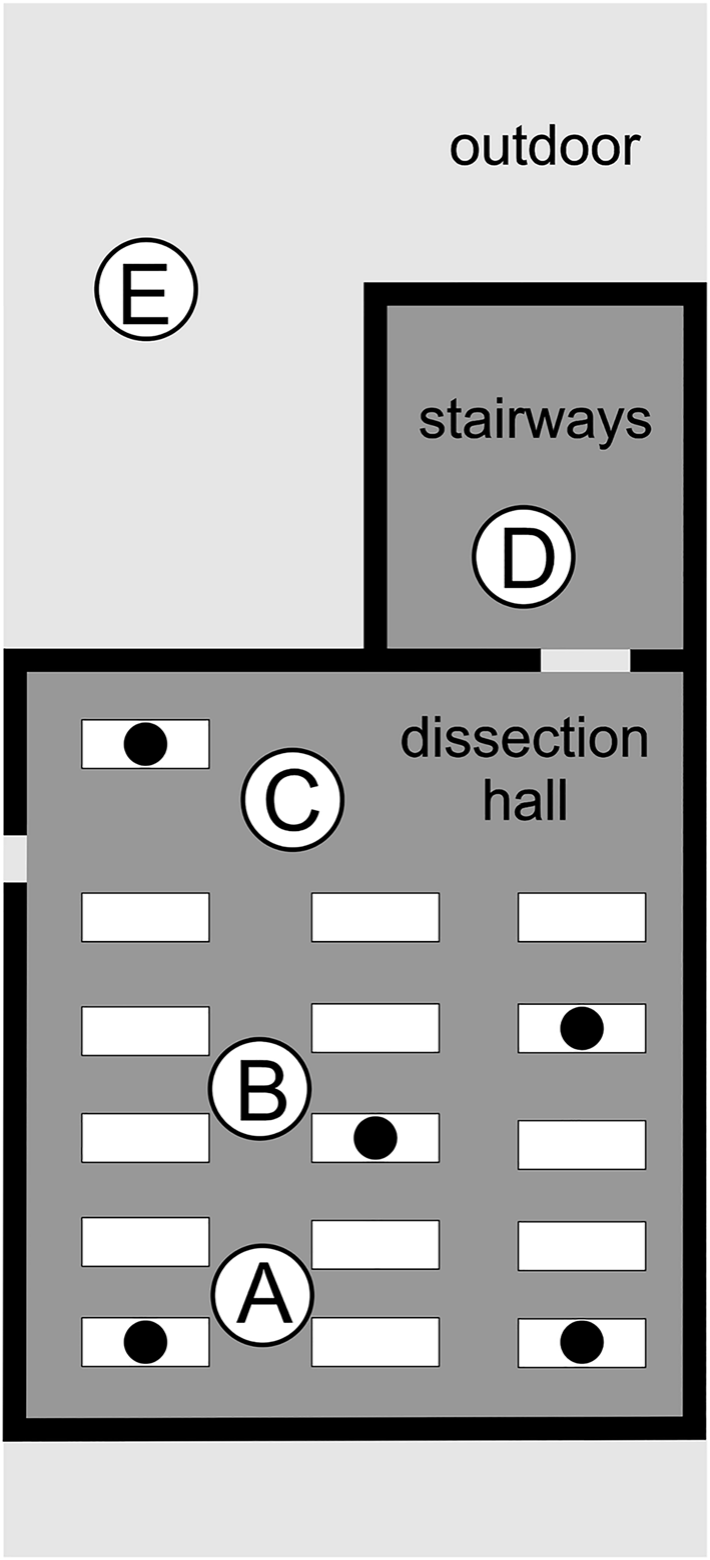
Sampling location for room air measurements (A-E) on site map of the anatomical dissection hall and adjoining stairways. (**A-C**) sampling locations with the dissection hall (2nd floor), (**D**) sampling location in the stairways (2^nd^ floor), (**E**) outdoor sampling location in the inner courtyard (ground-level) of the anatomical institute. Filled black circles indicate sites of tissue sampling from body donations.

Impaction-based air sampling was performed with a SpinAir air sampler (IUL Instruments, Barcelona, Spain). For the assessments, the air sampler was located on a tripod at a height of 1.3 m above the ground. The chosen device settings were as follows: duration 1 min, air volume 100 L, rotation speed of the agar plate 4/min. Air sampling comprised 5 sites of 3 different locations (Fig. 3):

- I. inside the dissection hall at 3 sites: near the windows wall (A), in the center (B), near the lecturer’s blackboard (C).
- II. at the stairway landing adjacent to (outside) the dissection hall (D).
- III. outdoors, at the inner courtyard of the institute (E).

The indoor measurement sites were located on the second upper level of the Anatomical Institute, while outdoor measurements took place at ground level, about 5 m from the entrance door. Throughout the measurement period, windows remained closed.

Depending on the time point, room air measurements in the dissection hall and the stairways adjacent to the dissection hall were done either during dissection times with students (i.e. “course”) or without students (i.e. “no students”).

Air temperature and humidity were measured for all locations. For each measurement, the room air was sampled on (a) Columbia agar (Becton Dickinson, Heidelberg, Germany), (b) ReadyPlate DG18 agar (Millipore, Darmstadt, Germany), and (c) malt agar (Millipore). Prior to each measurement date, the suction head was sterilized. Between the 5 different measurement locations (3 × dissection hall, 1 × stairways, 1 × outdoors), the suction head was disinfected with an alcohol-free wiper (Cleanisept Wipes Forte Maxi, Dr. Schumacher GmbH, Malsfeld, Germany).

Incubation times were (a) 2 days at 36 ± 1 °C for col agar plus additional 5 days at 20–25 °C, (b) 2 days at 36 ± 1 °C for DG18 agar plus additional 7 days at 20–25 °C, and (c) 2 days at 36 ± 1 °C for malt agar plus additional 7 days at 20–25 °C.

### Microbiological tissue sampling

For microbiological analyses, the following four tissue samples of approximately 4 mm^3^ each were collected from 5 body donations during the anatomical course on June 22nd 2023:

- Skeletal muscle (*Musculus sartorius*)
- Skeletal muscle (*M. rectus abdominis*)
- Liver (*Lobus caudatus*)
- Intestine (colonic mucosa)

Sample sites were chosen to cover different regions with varying microbiotic loads of the dissected corpses, including superficial sites (musculature) predominantly exposed to room air and abdominal sites with presumably sterile (liver) and non-sterile (colonic mucosa) tissues before embalming. Investigators wore surgical masks and disinfected gloves and used disinfected dissection instruments for each sampling. For bacterial analyses, samples were transferred into sterile 2 ml Precellys homogenization tubes with ceramic mix beads of 1.4/2.8 mm (Bertin Technologies, Montigny-le-Bretonneux, France) in 1 ml PCR water for homogenization with a Precellys Evolution homogenizer (VWR, Darmstadt, Germany) for 20 s at 7500 rpm as previously described^31^. Columbia and Schaedler agar plates (Becton Dickinson, Heidelberg, Germany) were inoculated with the different homogenates (100 µL each) and incubated at 36 ± 1 °C for 2 or up to 5 days under aerobic and anaerobic conditions, respectively. Colony forming units (CFU) were counted by macroscopic inspection of the agar plates, for aerobic incubated plates on day 1 and day 2, for anaerobic plates on day 3 and 5. Bacteria were identified by MALDI-TOF-MS using the MALDI Biotyper sirius IVD System (Bruker Daltonics, Bremen, Germany). Measurements were carried out according to the IVD-MALDI Biotyper standard procedure protocol with the MBT Compass IVD Software (v.4.3) utilizing the MBT IVD Library (v.11, revision G, 2021, Bruker Daltonics, Bremen, Germany).

For mold analysis, samples were transferred into sterile 2 mL tubes (Eppendorf GmbH, Wesseling-Berzdorf, Germany). Filamentous fungi were cultured on standard Sabouraud dextrose agar with gentamicin and chloramphenicol (Becton Dickinson, Heidelberg, Germany), and incubated at 28 ± 1 °C for up to 10 days. Fungal strains were identified firstly based on their macro- and micromorphological characteristics. Lactophenol cotton blue staining was used for microscopic examination of adhesive tape preparations from fungal cultures. All isolates were subjected to MALDI-TOF MS analyses using a Bruker MALDI Biotyper Sirius (Bruker Daltonics, Bremen, Germany). Sample preparation was performed by the Mycelium Transfer procedure (MBT HT Filamentous Fungi Module User Manual, Revision C). The resulting spectra were assessed by the MBT Filamentous Fungi Library Version 4.0. Species not clearly identified by MALDI-TOF MS or not included in the database, were evaluated by DNA sequencing (Microsynth Seqlab, Göttingen, Germany) of the D1-D2 region of the large subunit of the 28S ribosomal RNA gene^32^, the internal transcribed spacer (ITS1) region, β-tubulin, and calmodulin, respectively^33^.

### Statistics

GraphPad Prism version 8.4 was used to assess significant differences between sampling sites (GraphPad Software, San Diego, California USA). The non-parametric Kruskal–Wallis test for unequal group sizes was performed for the Analysis of Variance (ANOVA). For selected multiple comparisons between location we used Dunn’s post hoc test. Differences were considered significant at p ≤ 0.05. Results are presented either as total numbers or as mean ± standard deviation.

### Supplementary Information

Supplementary Information.

## Data Availability

The data that support the findings of this study are available on request from the corresponding author.
