# Supplementary material for: Quantitative and qualitative assessment of airborne microorganisms during gross anatomical class and the bacterial and fungal load on formalin-embalmed corpses
Source: Sci Rep. 2024 Aug 17;14:19061. doi: 10.1038/s41598-024-69659-y (PMC11330451; doi:10.1038/s41598-024-69659-y)
Supplement: Supplementary file 1 — Supplementary Information. [file 41598_2024_69659_MOESM1_ESM.pdf]

Supplementary Information

Title: A quantitative and qualitative assessment of airborne microorganisms during gross anatomical class and the bacterial and fungal-load on formalin-embalmed corpses

Authors: Jonas Keiler, Antje Bast, Jessy Reimer, Markus Kipp, Philipp Christoph Warnke

	mean CFU/m³				
bacterial species	outdoor [n=5]	dissection hall, course [n=6][1]	dissection hall, semester break (no students) [n=2][1]	stairways, course [n=6]	stairways, semester break (no students) [n=2]
Acinetobacter lwoffii	-	+	-	-	-
Arthrobacter koreensis	+	-	-	-	-
Arthrobacter pascens	+	-	-	-	-
Bacillus amyloliquefaciens	-	-	-	+	-
Bacillus cereus	-	+	-	-	-
Bacillus pumilus	-	+	-	+	-
Bacillus subtilis	-	+	-	+	-
Bacillus thuringiensis	+	+	-	-	-
gramlabile Bacillus with spore inclusions	-	+	-	-	-
Brevundimonas diminuta	-	+	-	-	-
Chryseobacterium hominis	-	-	-	+	-
Corynebacterium afermentans	-	+	-	-	-
Corynebacterium amycolatum	-	+	-	-	+
Corynebacterium aurimucosum	-	-	-	-	+
Corynebacterium gottingense	-	+	-	+	-
Corynebacterium species	-	-	+	-	-
Corynebacterium tuberculostearicum	-	+	-	+	+
Dermabacter hominis	+	-	-	-	-
Dermacoccus nishinomiyaensis	-	+	-	+	-
Exiguobacterium artemiae	-	-	-	+	-
Exiguobacterium sp	-	+	-	-	-
Haematobacter massiliensis	-	+	-	-	-
Kocuria carniphila	-	+	-	-	-
Kocuria rhizophila	-	+	-	+	-
Kocuria rosea	-	+	+	+	-
Microbacterium paraoxydans	-	-	-	+	-
Micrococcus cohnii	-	+	-	-	-
Micrococcus luteus	-	+	+	+	-
Micrococcus terreus	-	+	-	+	-
Micrococcus species	+	-	-	-	-
Moraxella osloensis	-	+	+	+	-
Neisseria flavescens	-	+	-	-	-
Neobacillus drentensis	-	+	-	+	-
Niallia circulans	-	+	-	-	-
Oerskovia turbata	-	+	-	-	-
Paenibacillus amylolyticus	-	-	-	+	-
Pantoea agglomerans	-	-	-	+	-
Pantoea eucrina	-	+	-	-	-
Pantoea septica	-	+	-	-	+
Paracoccus yeei	-	+	-	+	+
Peribacillus simplex	-	+	-	-	-
Priestia megaterium	+	-	-	+	-
Pseudarthrobacter oxydans	+	-	-	+	-
Pseudarthrobacter scleromae	+	-	-	+	-
Pseudomonas oryzihabitans	-	+	-	-	-
Roseomonas mucosa	-	+	-	-	-
Rothia dentocariosa	-	+	-	-	-
Sporosarcina ureae	-	+	-	-	-
Staphylococcus aureus	-	+	-	-	-
Staphylococcus borealis	-	+	-	-	-

Staphylococcus capitis	-	+	+	+	-
Staphylococcus caprae	-	-	-	+	-
Staphylococcus cohnii	-	+	-	-	-
Staphylococcus condimenti	-	-	-	+	-
Staphylococcus epidermidis	-	+	-	+	-
Staphylococcus haemolyticus	+	+	-	+	-
Staphylococcus hominis	-	+	+	+	-
Staphylococcus pettenkoferi	-	+	-	-	-
Staphylococcus pragensis	-	+	-	-	-
Staphylococcus saprophyticus	-	+	-	+	-
Staphylococcus succinus	-	+	-	+	-
Staphylococcus warneri	-	+	+	-	-
Streptococcus mitis oralis	-	+	-	-	-
Streptococcus vestibularis	-	+	-	-	-
Viridibacillus neidei	-	+	-	-	-

Supplement S1. Presence of detected bacterial species for sampling locations. [1]Presence for the measured locations in the dissection hall was pooled.

fungal species	outdoor [n=5]	dissection hall, course [n=6][1]	dissection hall, no students [n=2][1]	stairways, course [n=6]	stairways, no students [n=2]
Penicillium citrinum	-	-	-	-	+
Penicillium citreonigrum	+	-	-	-	-
Penicillium griseofulvum	+	-	-	-	-
Penicillium chrysogenum	-	-	-	+	-
Penicillium commune	+	-	-	-	-
Penicillium atrosanguineum	+	-	-	-	-
Aspergillus niger	+	-	+	+	+
Aspergillus fumigatus	+	-	-	+	-
Aspergillus montevidensis	+	-	-	+	-
Aspergillus puulaauensis	+	-	-	-	+
Alternaria abundans	-	-	-	-	+
Alternaria rosae	+	-	-	-	-
Alternaria alternata	+	-	-	+	-
Alternaria infectoria	+	-	-	+	-
Talaromyces sp. (columbinus)	-	-	-	-	+
Talaromyces sp. (wortmanni)	+	-	-	-	-
Mollisia hydrophila	+	-	-	+	-
Agrocybe praecox	-	-	-	+	-
Purpureocillium lilacinum	-	-	-	+	-
Ustilago filiformis	+	-	-	-	-
Arthrinium sp.	-	-	-	+	-
Neurospora tetrasperma/sitophila	+	-	-	-	-
Fomitopsis pinicola	-	-	-	+	-

Supplement S2. Detected fungal species in the different locations. [1]Presence for the measured locations in the dissection hall was pooled.
